# Riverbank filtration: a frontline treatment method for surface and groundwater—African perspective

**DOI:** 10.1007/s10661-024-13413-4

**Published:** 2025-01-10

**Authors:** Fasilate Uwimpaye, Gratien Twagirayezu, Isaac Odiri Agbamu, Karolina Mazurkiewicz, Joanna Jeż-Walkowiak

**Affiliations:** 1 Institute of Environmental Engineering and Building Installations, Faculty of Environmental Engineering and Energy, Poznan University of Technology, Berdychowo 4, 60-965 Poznan, Poland; 2 State Key Laboratory of Environmental Geochemistry, Institute of Geochemistry, Chinese Academy of Sciences, 550002 Guiyang, Guizhou China; 3 University of Chinese Academy of Sciences, Beijing, 100049 China; 4 Faculty of Civil Engineering, Geodesy, and Transport, Institute of Building Engineering, Poznan University of Technology, Piotrowo 5, 60-965 Poznan, Poland

**Keywords:** Water treatment, Riverbank, Filtration, Contaminants, Surface water, Groundwater, Africa

## Abstract

Riverbank filtration (RBF) has emerged as a crucial and functional water treatment method, particularly effective in improving surface water quality. This review is aimed at assessing the suitability of RBF in regions with limited access to clean water, such as Africa, where it has the potential to alleviate water scarcity and enhance water security. This review used various studies, highlighting the principles, applications, and advancements of RBF worldwide. The findings of this review revealed that RBF effectively addresses a broad range of contaminants, including microbial pathogens, organic compounds, heavy metals, and micro-pollutants, through natural processes like adsorption, biodegradation, and filtration. These natural mechanisms significantly reduce waterborne contaminants, making RBF an eco-friendly, sustainable, and cost-effective alternative to conventional water treatment methods. Hydro geological factors, such as aquifer thickness and hydraulic conductivity, play an important role in the efficiency and overall performance of RBF systems. The integration of RBF with advanced treatment technologies not only removes contaminants more effectively but also ensures a sustainable supply of clean water for various applications. The cost-saving aspect of RBF, compared to traditional methods, is particularly significant in low-income regions. The study suggests a wider use of RBF, particularly in Africa, where it can strengthen resilient water supply systems in response to growing water scarcity and climate change concerns.

## Introduction

With the development of industry, agriculture, and modern lifestyles, dangerous and toxic substances are released into the environment and surface water sources. This pollution poses a serious challenge to the drinking water supply of industrial and agricultural activities. Rivers and lakes that serve as drinking water sources and habitats for aquatic life are particularly at risk of pollution from organic and inorganic substances released by various sectors. Examples include the Ganges (India), one of the most polluted rivers in the world due to agricultural runoff, raw sewage, and industrial wastewater. An estimated 600 million people rely on this river for drinking water, and pollution affects their health and aquatic life (Lal, 2019). This contamination only increases with urbanization and industrialization. Unfortunately, most conventional water treatment facilities struggle to remove increased levels of physicochemical and bacteriological contaminants in surface water, including emerging contaminants like pharmaceuticals, personal care products, and surfactants (Sandhu et al., 2019; Naidu et al., 2021). Consequently, introducing a natural, reliable, cost-effective, and robust water treatment method called River Bank Filtration—RBF—may be the best purification option for water treatment (Abdelrady et al., 2020). It is crucial to tackle those problems by providing quantity and quality drinking water that meets the standards and saves lives worldwide (Dragon et al., 2019; van Driezum et al., 2019).

RBF has become a significant water treatment method, spanning over a century in Europe and several decades in the USA (Gillefalk et al., 2018). Moreover, its use has expanded to wealthy developing nations, including Egypt (Wahaab et al., 2019), China (Pan et al., 2018), Korea (Maeng & Lee, 2019), India (Boving et al., 2018), and Brazil (Romero-Esquivel et al., 2017). RBF can be defined as a process by which surface water, often from rivers or lakes, infiltrates aquifers through riverbanks. This water is then collected by collecting wells and transported for further treatment (Gillefalk et al., 2018; Patenaude et al., 2020). Various mechanisms, including physicochemical and bacteriological, come into play during this subsurface passage, leading to the reduction or removal of contaminant concentrations. RBF can serve as a standalone water treatment method and a preliminary treatment stage (Regnery et al., 2015). Its application depends on various factors, including the intended purpose of water use and the required extent of filtration and contaminant reduction. Depending on specific objectives, RBF can be used with advanced treatment techniques such as oxidation, ion exchange, adsorption, soil aquifer treatment, and membrane filtration (Ghodeif et al., 2016).

Simultaneously, RBF, with its extensive historical use and global reach, represents a versatile surface water treatment adaptable to various contexts and integrated into advanced treatment strategies based on specific water quality objectives. Numerous aquifer processes can improve surface water quality during riverbank filtration. Accordingly, a series of biological, chemical, and physical processes (Ghodeif et al., 2018) called filtration, ion exchange, complexation, solution-precipitation, sorption–desorption, microbial biodegradation, redox reactions, and dilution of infiltrated water have gone through these natural processes, thus significantly improving water quality by removing or reducing heavy metals, organic compounds, bacteria, pathogens, and viruses (Masse-Dufresne et al., 2019). During the filtration process, biodegradation and adsorption play a role in eliminating dissolved and suspended contaminants, along with pathogens, present in infiltrated water (Bertelkamp et al., 2014), because as the surface water percolates through the soil and into wells, it passes through a natural filter, which can improve its quality. The hydrogeological and hydrological conditions of the surface water and groundwater have a major impact on the effectiveness of RBF.

Before implementing RBF in any region, a thorough evaluation of its suitability and effectiveness is required. Scientists, engineers, and researchers should consider several factors that can affect RBF performance. These factors include the pollutant load in the body of water, the composition of bank materials (including the quantity of sand, stones, gravels, clays, and their distribution), the depth and distance of the water abstraction device, the abstraction rate, seasonal fluctuations in rainfall and water levels, and region-specific geological and groundwater flow characteristics (Kumar et al., 2018). However, a standard protocol has yet to be found as it is site-specific technology. Hiscock and Grischek (2002) revealed that surface water quality influences redox conditions that raise the concentrations of dissolved manganese, ammonium, hydrogen sulfide, and iron in drinking water. The study by Hiscock and Grischek (2002) exposed that surface water quality impacts redox conditions, increasing dissolved iron, ammonium, hydrogen sulfide, and manganese concentrations in drinking water.

Indeed, the RBF system is significantly affected by the quality of surface water. Therefore, an in-depth understanding of reciprocity is essential to ensure the future implementation of this technology in drinking water supply systems. Ultimately, this study aimed to evaluate the feasibility of the RBF method in improving water quality by reducing contaminants and pathogens. Moreover, the target here is to assess its efficiency and effectiveness in treating water from various sources, including surface water and groundwater, therefore making it suitable for various purposes such as drinking water supply, industry, and irrigation.

Around the world, many countries, as well as Africans, are facing problems related to clean water. However, the capacity for distributing drinking water is multiple times less than required (Shebl et al., 2022). Human and natural factors include urban development, poverty, population spread, rainfall deficit and droughts, and increased water demand, which are major contributors to water shortage (Matchawe et al., 2022). Africa is the home of 50% of the world’s population who lacks clean water for drinking (WHO & UNICEF, 2015). Bank filtration can be a promising solution to reduce water scarcity due to its safety, sustainability, efficiency, and cost-effectiveness in water treatment. Most African countries have not implemented a natural water treatment technique called riverbank. With cautious preparation, adaptation, and restoration to local scenarios, bank filtration can secure water and boost African resilience (Ebrahim et al., 2020). The operational cost of existing water treatment methods exceeded the bank filtration system. Thus, bank filtration may be considered water storage, allowing better water management in the absence of a continuous supply of natural raw water in developing nations (primarily African) and used as a fundamental treatment stage (Sharma et al., 2012) or as the first barrier of treatment approach (for example, followed by membrane filtration in high-income countries).

The goal of the study is to evaluate the suitability of RBF in areas with restricted access to clean water, like Africa, where it could help reduce water scarcity and improve water security. It also examines the effectiveness of RBF in improving water quality by reducing contaminants. In addition, it explores the economic viability of implementing RBF systems in the communities and evaluates the potential environmental impacts of RBF on local ecosystems. Furthermore, it identifies the challenges and barriers to the widespread adoption of RBF in the regions.

## Riverbank filtration water intake—fundamental issues

RBF sites globally present variations in structure and function, shaped by their specific treatment objectives. The design of these sites involves a complex set of processes and factors that need to be carefully considered. Water quality and quantity are the primary parameters when designing a riverbank filtration system. This means that the intake should provide enough water at an acceptable quality to meet the intended purpose (Ray, 2008). The nature of sediments between the intake and surface water source, along with the distance between the abstraction point and the water source, plays a vital role in assessing the quantity and quality of water in the system. Hydrogeological conditions, including the connection between river and aquifer, the thickness of the aquifer, hydraulic conductivity, and decreased river bed clogging as a result of the erosive flow of a river for a particular area, can make RBF applicable or favorable. Fundamental issues are as follows: the selection of filtration locations along the riverbanks and the feasibility study, technical and operation aspects of RBF, and the assessment of water quality during filtration along the riverbanks.

It should be noted that clogging at the interface between surface water and groundwater can significantly affect intake rates. In particular, biological clogging can interfere with riverbank filtration systems (Dubuis & De Cesare, 2023; Gaol et al., 2021). This happens when microorganisms grow and form biofilms in sediments, reducing their permeability and impeding water flow. Over time, the accumulation of organic material can significantly reduce filtration efficiency and require intervention to restore system performance.

### Riverbank filtration site selection and feasibility study

**Fig. 1:**
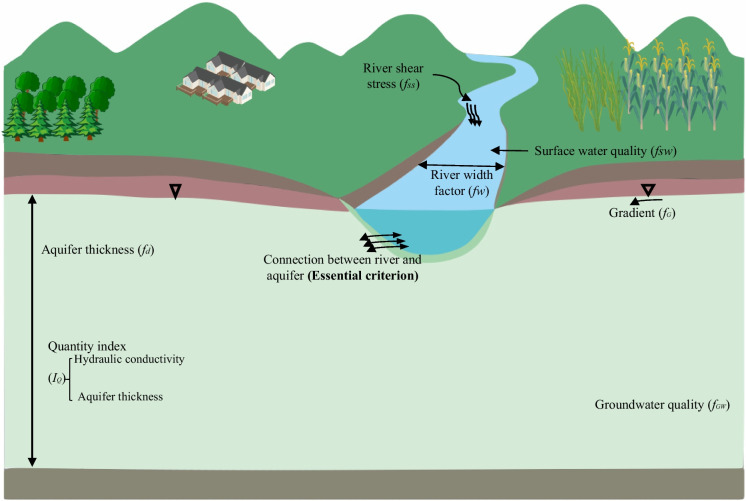
Criteria for RBF site selection

The study carried out by Wahaab et al. (2019) demonstrated that the efficiency of the RBF system is primarily dependent on the placement of the Bank Filtration (BF) wells. Nevertheless, it is a technique specific to a particular site’s characteristics and conditions. As indicated in Fig. 1 (Hoang et al., 2022), a multicriteria of site evaluation is typically conducted to determine the ideal site for the RBF system. The evaluation process is divided into three stages as follows: firstly, the correlation check between the river and aquifer, regarded as a critical criterion by various authors such as Hoang et al. (2022) and Patil et al. (2020), in determining the suitability of the site; secondly, assessing the aquifer storage potential; and thirdly, determining the quantity of bank filtrate that could be efficiently extracted through a set of quantity criteria. Finally, the surface and groundwater quality are assessed as water quality indicators. The result of the quantity and quality evaluation is stated as a site-suitable index (SSI), which extends from “0” to “1.”

A higher index indicates a higher suitability. However, a site with remarkably low transmissivity is still unsuitable for RBF application, although it has a high index. This is due to the need to meet additional criteria, such as the following: ensuring sufficient hydraulic conductivity necessary to achieve the desired intake performance. In the case of a rapid decrease in the transport of infiltrated water during the operation of an infiltration water intake, the cause may be the accumulation of sediments or the formation of biofilm. Ensuring adequate intake capacity requires additional work, i.e., stirring up the river bottom or cleaning the bottom of infiltration ponds (in the case of artificial infiltration with recharge of river water to the ponds before the abstraction of water from the well). Other factors include ensuring the quality of surface water and groundwater and maintaining a gradual slope between the river and aquifer. These factors are essential to guarantee the appropriateness of the site for the application of RBF.

**Fig. 2:**
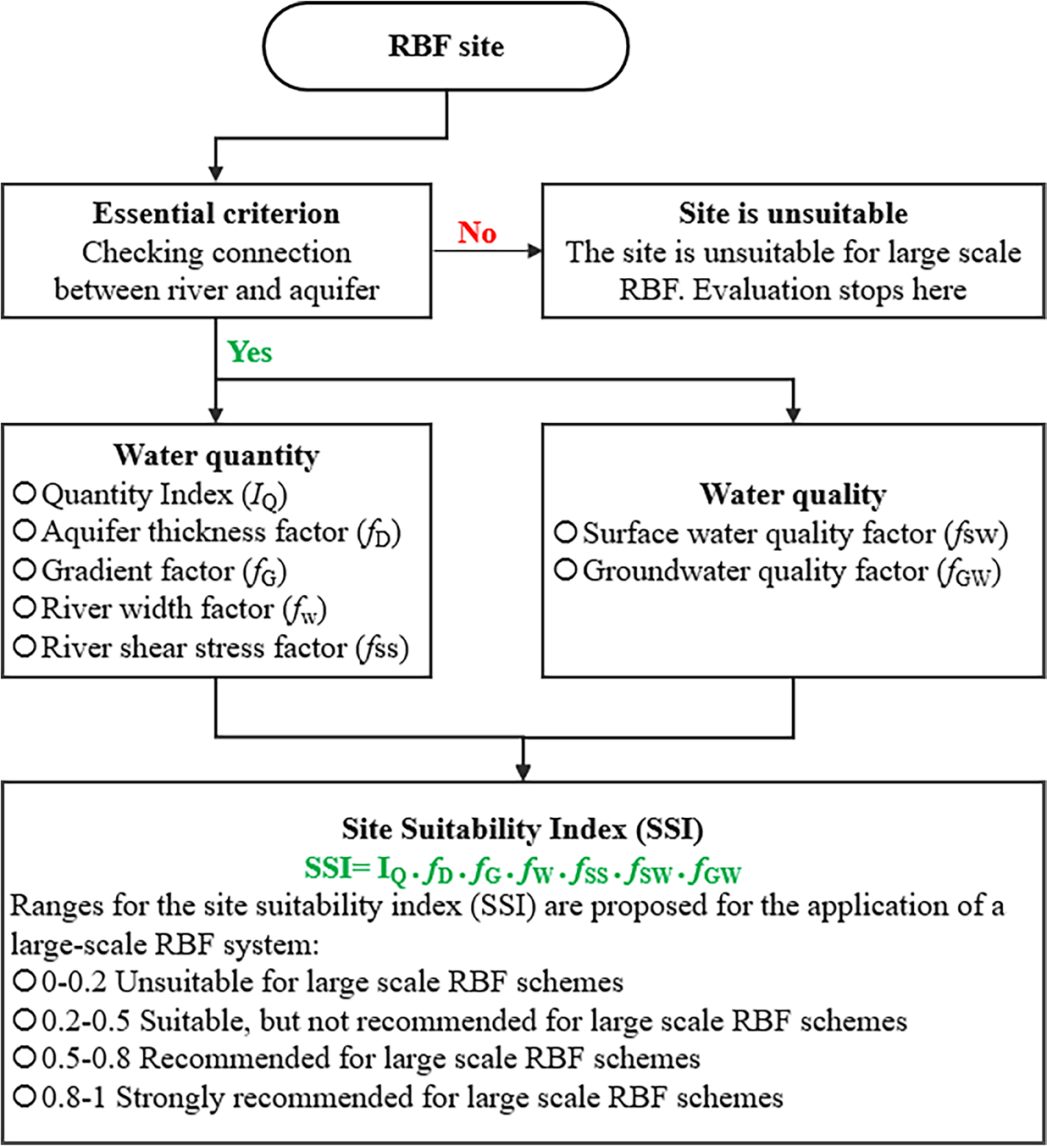
Flow diagram for the methodology application

Bartak et al. (2015) proved that conducting a study on a small scale at the site can be costly and constrained, primarily due to the requirements of procedures such as drilling, borehole logging, pumping tests, and prolonged hydro-chemical sampling and analysis. Therefore, conducting site investigations across extensive areas is crucial, as is using comprehensive databases and implementing multicriteria analysis during the evaluation process. In areas where the implementation of RBF is not common, a notable advantage of incorporating it into the water distribution network can be observed. As shown by Srisuk et al. (2012) and Patil et al. (2020), the main criterion for selecting a site for the RBF system is the hydraulic linking between the aquifer and river, as illustrated in Fig. 2 (Hoang et al., 2022). Unless the majority of water withdrawn consists of groundwater rather than bank filtrate, this criterion should be included in any RBF site selection method used, regardless of the application area or its intended use. As reported by Hoang et al. (2022), a site requiring a hydraulic connection between the river and the aquifer is promptly dismissed or denied. Nevertheless, the selection of sites for RBF systems depends on their applicability. For instance, when the objective of RBF is to extract significant amounts of water, an affordable location can feature an aquifer that permits substantial abstractions.

The quantity of water extractable from an aquifer is primarily contingent on two crucial factors: hydraulic conductivity (K) and aquifer thickness (D). These factors collectively contribute to the aquifer transmissivity (T). Generally, regions characterized by high aquifer transmissivity facilitate increased water abstraction rates, whereas areas with low transmissivity tend to have lower rates of water movement. However, even in low transmissivity aquifers, increasing the number of wells can still help RBF achieve high abstraction rates. One downside of RBF is that it requires extensive and expensive areas, making it less practical for some applications. Alluvial aquifers where RBF systems are constructed are usually located ahead of riverbanks. The soil texture of the aquifer can be composed of different classes of deposition ranging from fine to coarse particles. In the context of the RBF system, conditions defined by a coarse-in-texture aquifer, which indicates permeable water-bearing deposits that are hydraulically coupled with riverbed materials, are also favorable.

Additionally, the costs of using RBF with low transmissivity can be prohibitive. Considerations such as RBF system design (pumping rate, well count, and miles within wells and the riverbank) and site characteristics (aquifer thickness, groundwater gradient, river course, and clogged riverbed) affect the volume of water abstracted. Moreover, increased levels of clogging can significantly reduce the effectiveness of RBF, emphasizing the importance of avoiding sites with a high clogging potential.

**Fig. 3:**
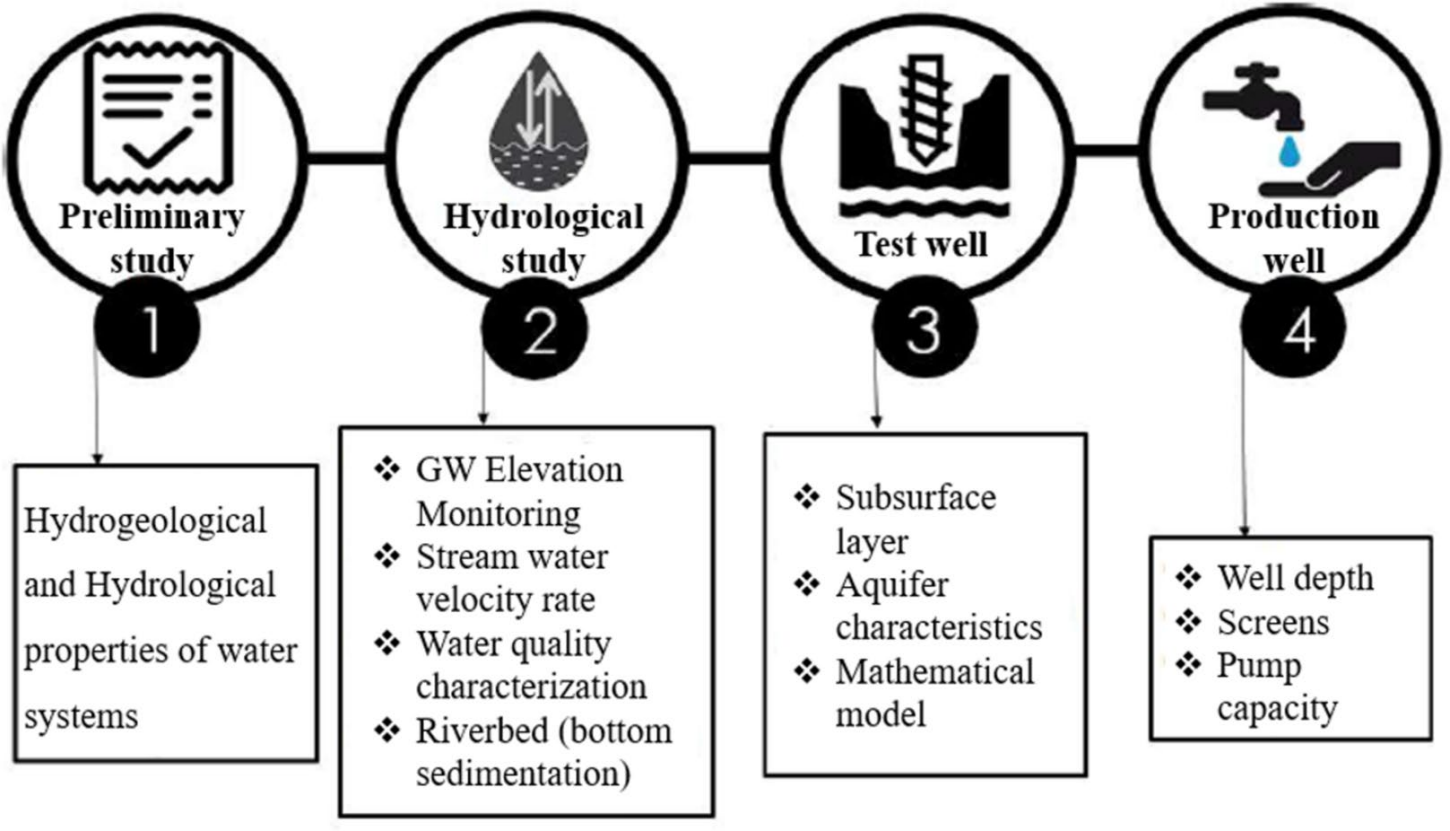
Four-stage investigation plan for site selection

Indicators of low clogging potential at sites include elements such as high river shear stress, a substantial area of river infiltration, and water of excellent or superior quality that has been infiltrated (Grischek & Bartak, 2016; Hubbs, 2006; Schubert, 2002). Only a few researchers considered assessing clogging when choosing a site (Patil et al., 2020; Wang et al., 2016). Since the primary objective of RBF is to improve water quality when evaluating site selection, it is critical to consider the surface and groundwater quality—the process of selecting a suitable site for riverbank filtration (RBF), as proposed by Hagras (2021) in Fig. 3 which involves the following four stages: (a) preliminary study on the hydrological and hydrogeological properties of the water, (b) identification of an appropriate site for suitable placement, (c) determination of the hydrological parameters of the aquifer, as well as the monitoring of the characteristics and levels of surface water, and (d) creation of a computational model to predict travel time and to determine the percentage share of groundwater filtrate in the pumped water.

**Fig. 4:**
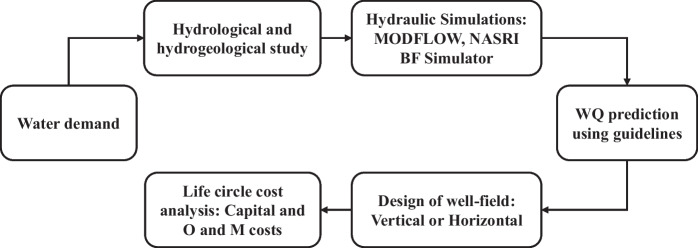
A framework for assessment of the feasibility of RBF technology at a particular location

For riverbank filtration to be viable, several crucial factors must be considered, such as the following: hydrogeological and hydrological situations, surface and groundwater quality, and the associated costs of capital, operations, and maintenance. Figure 4 highlights essential aspects that require an assessment to evaluate the feasibility of RBF (Sharma et al., 2012). The feasibility of RBF can be influenced by various factors related to surface water, such as flow rate, water level fluctuations, and sediment transport. For RBF to succeed, the river flow must remain stable and continuous throughout the season to ensure a constant water supply. Water level fluctuations and sediment transport can negatively affect the quality of percolating water and lead to a sediment buildup in the riverbank and aquifer, further impacting RBF. Therefore, it is essential to conduct thorough hydrological studies that include long-term monitoring to assess the appropriateness of surface water for RBF. Surface water levels can be lowered under steady-state conditions using software like MODFLOW Pro (PMWIN Pro). However, the software requires information from hydrogeological and hydrological parameters, including flow to determine the pumping yield (flow rate) table, ground layers, hydraulic conductivities, aquifer depth, aquifer type, and porosities. Hence, MODFLOW obtained the number of wells, their spacing, and the traveling time required under steady-state conditions.

In addition, as several researchers have pointed out (Holzbecher, 2006; Holzbecher & Sorek, 2005), the NASRI Bank Filtration Simulator (version 1.3a) has been used in Germany as part of the Natural Systems for Recharge and Infiltration projects to determine the percentage of bank filtrate and local groundwater in the vicinity of a particular well system. Indeed, calculating the proportion of bank filtrate at a particular well location involves considering different factors, such as hydraulic conductivity, base flow, extraction rates, distance to the river, spacing between wells, and the total number of wells. Assessing the quality of water filtered from riverbanks is critical to this process. The efficiency of removing RBF is generally determined by the residence time and traveling distance of surface water to the well. Investigators use natural filters and a mixture of local groundwater effects to identify specific contaminant removal and determine the ultimate water quality. The following mass balance equation below is used to predict the top water quality:

$${C=C}_{\text{river}}*{P}_{\text{RBF}}*\left(1-{R}_{\text{RBF}}\right)+{C}_{\text{GW}}*(1-{P}_{\text{RBF}})$$

where *C* = contaminant levels in the treated water from the bank filtration system, *C*_river_ = content of a contaminant in the water surface, *R*_RBF_ = the contaminant removal effectiveness of the bank filtration system, as per the established guidelines, *P*_RBF_ = percentage share of bank filtrate (proportion of bank filtrate as a percentage), *C*_GW_ = contaminant level in the indigenous groundwater.

When the required parameters for RBF are in place, it becomes feasible to estimate all correlated expenses. These expenses include the capital costs of constructing RBF, including labor, wells, pumps, power, and other treatment facilities. Additionally, operation and maintenance costs and potential revenue from water sales must be taken into account. A comprehensive economic analysis is necessary to determine whether RBF is cost-effective compared to conventional water treatment. To accurately determine RBF costs, various factors must be considered such as attributes of the aquifer, well-screen installation type, the layout of the project, and proximity to the beneficiary communities. Ray et al. (2002) declared that RBF is not only worthy for water treatment nor cost-effective but also an environmentally friendly and long-term solution for posterity. Hunt and Stabeno (2002) discovered that the dimensions of the bank filtration system, the varieties of wells, the frequency with which they operate irregularly, the materials used to build the wells, the surrounding geology, and the state of the river all play a role in the operating and maintenance expenses. Finally, Fig. 4 and Table 1 show the hydrogeological information used in MODFLOW simulations and the main aspects of water supply examined for the feasibility of RBF in two African countries (Malawi and Kenya).

**Table 1:** Examples of data used during the feasibility of RBF for different cities in Malawi and Kenya

Description	Blantyre	Lilongwe	Mzuzu	Eldoret	Nakuru
Mesh size (m)	5000 × 5000	5000 × 5000	5000 × 5000	7500 × 6000	5000 × 5000
Number of rows	200	200	200	250	200
Number of columns	200	200	200	300	200
Aquifer type	Unconfined	Unconfined	Unconfined	Unconfined	Unconfined
Main aquifer material	Silty gravelly sands	Gravelly clayey sands	Clayey, silty sands	Coarse gravel and silty clay	Sandy sediments
Aquifer thickness (m)	30	50	40	50	60
Average hydraulic conductivity (m/s)	5.7 × 10^−5^	3.9 × 10^−5^	2.9 × 10^−5^	6.0 × 10^−4^	1.66 × 10^−3^
Average porosity	0.29	0.28	0.24	0.28	0.28
Recharge rate (m/s)	5 × 10^−10^	2 × 10^−9^	2.7 × 10^−9^	4.1 × 10^−9^	3.2 × 10^−8^
Population	700,000	670,000	135,000	217,000	285,000
Water sources	Shire river, Mudi dam	Lilongwe river	Lunyangwa river	Ellegirini, Endoroto and Moiben rivers	Lake water (31%); Groundwater (69%)
Existing water treatment plant capacity (m^3^/day)	110,000	75,000	15,000	22,000	35,950
Operational costs of existing treatment system (US $/m^3^)	0.120	0.030	0.009	0.043	0.029
Distance of the wells from the bank (m)	50	50	50	100	50
Well-placing center to center (m)	100	100	100	50	50
Share of bank filtrate in proposed BF system (%)	83	88	89	95	91
Estimated operational costs of BF system (US $/m^3^)	0.098	0.014	0.009	0.036	0.020

In addition, the results of the study conducted by Shebl et al. (2022) indicate that the RBF capacity along the Nile and its branches in Egypt removed turbidities and microorganisms before they entered the intake well. Sharma et al. (2012) and Grischek and Bartak (2016) confirmed hydrogeological condition sand operating costs favor RBF in Africa, specifically Egypt, Malawi, and Kenya. Examples of limitations of applying RBF in Africa are awareness and familiarity, financial means, the potential of individuals and organizational units, and comprehension of the hydrogeology and geochemical characteristics of aquifers.

### Technical and operation aspects of infiltration water intake

#### Types of infiltration water intakes

There are two types of infiltration water intakes: bank filtration (RBF) and artificial infiltration (AIN). In fact, RBF is a process of natural filtration of water from a river or other surface water bodies through the bank or subsurface sediments. Ray et al. (2003) stated two types of riverbank filtration. The first is vertical bank filtration, which uses vertical wells located near the riverbank, allowing water to infiltrate vertically through sediments before being collected at the wells. This method is often applied when geological conditions favor vertical water flow. The second is horizontal bank filtration, where horizontal drains positioned below the riverbed collect water from a series of wells near the water source. Both methods effectively remove suspended particles, pathogens, and dissolved contaminants while at the same time improving water quality. The RBF intake is built close to rivers, which allows groundwater resources to be regularly supplemented with surface water. This type of infiltration is sometimes called natural. It occurs when the natural water table is above the groundwater level, while there is a possibility of infiltration of surface water into the aquifer. Natural infiltration can be used to increase the efficiency of groundwater intake.

In the artificial infiltration (AIN) process, artificial infiltration ponds are recharged with surface water, and the water infiltrates the collecting wells through the bottom of the pond and further through the ground. Artificial infiltration is used for more polluted surface water. As a result of the initial treatment of surface waters, the pond bottoms become clogged with sediments, which reduces the filtration efficiency. Therefore, it is necessary to provide periodic treatment. The ponds that are part of AIN intakes also offer storage for surface water, allowing the intake to operate in the event of pollution in the river that supplies it.

These two types of infiltration—RBF and artificial—often occur together in a single intake and are the record of the history of increasing intake capacity in developing cities. First, the groundwater intake is enriched by water infiltrating through the river bottom into wells located along its bank. Further expansion often requires the construction of infiltration ponds into which water is pumped from the river, and infiltration takes place in the ground, going through the bottom of the pond to a collecting well.

#### Wells

**Fig. 5:**
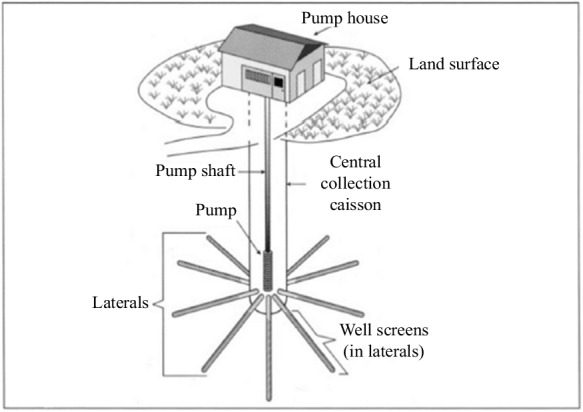
Horizontal or radial collector well with a pump house (Chittaranjan Ray et al., 2003)

**Fig. 6:**
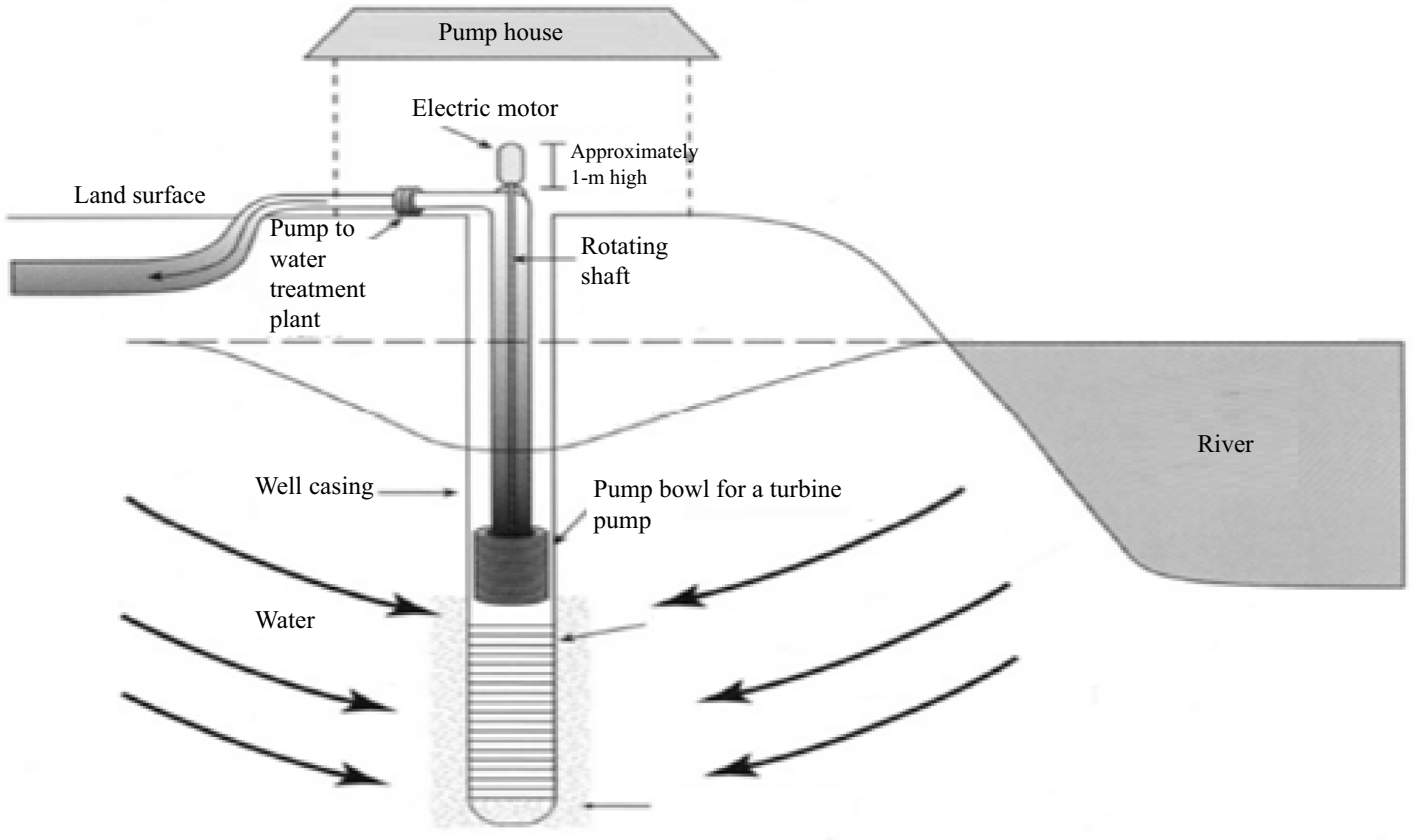
Vertical well with a pump house (Chittaranjan Ray et al., 2003)

**Fig. 7:**
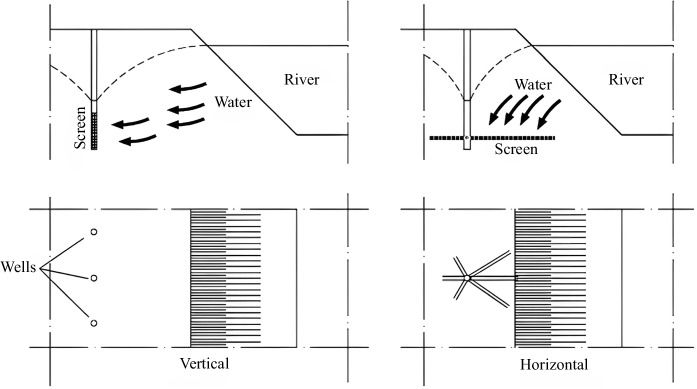
Vertical and horizontal wells correlate with river water (Chittanranjan Ray et al., 2002)

**Fig. 8:**
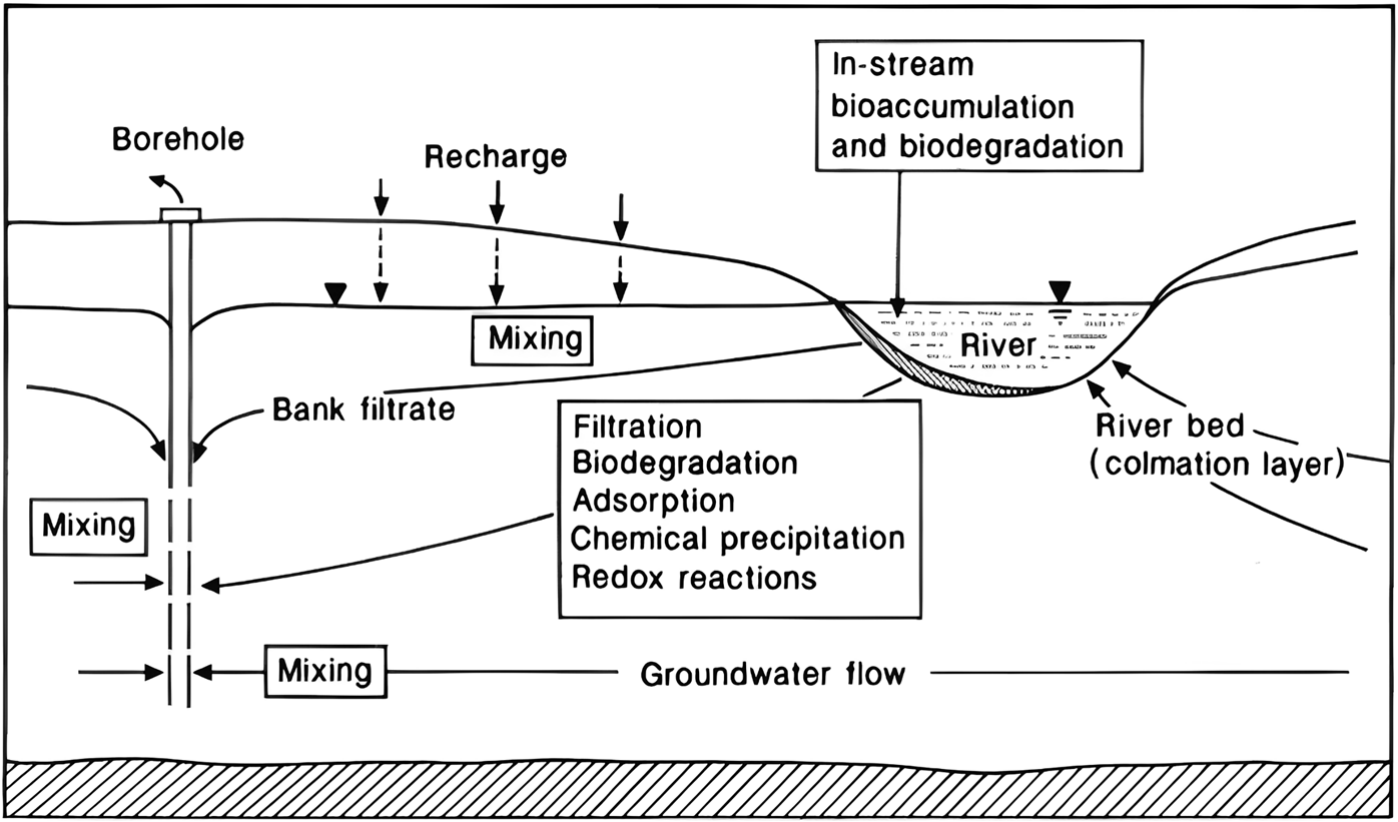
Schematic diagram of Riverbank filtration processes (Grischek & Paufler, 2017)

Wells dug into the ground are structures designed to extract water from aquifers. Proper location and construction are critical to ensure the quality and quantity of water available. When conducting the RBF procedure, carefully considering the distance between the wells and adjacent riverbanks is essential. An evaluation with a detailed analysis of the strengths and weaknesses of the RBF process is necessary to achieve the best design, as noted by Schijven and Hassanizadeh. (2000). Two types of wells can be classified based on their location on the riverbank: horizontal and vertical, and their comparison can be found in Table 2 (Hagras, 2021). A horizontal or radial collector well features aligned pipes that connect to a vertical collector pipe. Water collected by a horizontal well has passed through the aquifer. Typically, a radial collector well is constructed near the river using pipes under the riverbed to collect water directly into the well, as illustrated in Figs. 5, 6, and 7. However, the riverbank filtration processes are shown in Fig. 8 (Grischek & Paufler, 2017).

**Table 2:** Comparison between vertical and horizontal wells (Hagras, 2021)

	Vertical well	Horizontal collector well
Depth	5–100 m	< 40 m
Capacity	< 200 m^3^/h	1000–4000 m^3^/h
Advantages	Easy to construct (standard)	Less space needed
Disadvantages	Limited capacity	Special company for construction

The increase in the volume of water generated by the RBF process depends on various factors, including the healthy screen characteristics, porous media properties, layout, and the scope of lateral pipes in the radial collector well (Lee et al., 2012). All this shows that proper well design can make the RBF process easier and more efficient. For several reasons, a horizontal well is considered more efficient and preferable than a vertical one. It reduces the water level drop, maintains a lower velocity after entering the pipes, and requires fewer cleaning operations, resulting in lower operating costs (Bakker et al., 2005), and is the only possibility of limited screening intervals for vertical wells (Hunt & Stabeno, 2002). Some RBF wells are designed to rely directly on the natural water stream to transfer surface water into the pumping well, whereas others use screens to collect water from the surrounding aquifer (Ray et al., 2002).

Operating a bank filtration well involves a number of important issues. First and foremost, the well should be appropriately designed and constructed to adapt to the specific needs of the site, allowing continuous operation 24 h a day, 7 days a week. According to Grischek and Ray (2019), the location and number of wells can be considered to maximize water quality and quantity of bank filtrate. A regular maintenance schedule should be established to inspect and clean the well and its components to prevent significant sediment or biofilm buildup that can cause low water production (Grischek & Ray, 2019). Continuous operation is necessary to meet water demand and ensure proper well management to prevent issues threatening the system’s efficiency (Kuehn & Mueller, 2000). These steps are required to ensure the long-term reliability and performance of bank filtration wells.

#### Riverbed and pond clogging

Riverbed clogging is a critical factor in assessing the viability of infiltration systems. Clogging of the riverbed and pond bottom has positive and negative effects on water infiltration. It encourages the decomposition of pollutants by microorganisms but also reduces the hydraulic conductivity of underground reactions during the purification process. Clogging occurs when suspended organic and inorganic materials aggregate or deposit within a sediment matrix. This ultimately leads to a reduction in the capacity of the sediment matrix to allow for infiltration. According to Hutchison et al. (2013), clogging might have a physical, mechanical, chemical, and biological origin. Physical clogging is associated with inorganic and organic suspended solids. Biological clogging is caused by the accumulation and growth of bacterial cells in the bottom of a river or infiltration pond (Baveye et al., 1998; Engesgaard et al., 2006; Pavelic et al., 2011). The growth of these bacterial cells is dependent on physical–chemical factors associated with both soil and water (Pavelic et al., 2011). Mechanical clogging refers to air entrainment during recharge from the vadose zone to the phreatic aquifer or gas binding due to microbial activity (e.g., methane) (Jeong et al., 2018). Chemical clogging is the loss of hydraulic conductivity of the streambed due to the precipitation of initially soluble water constituents because of changes in redox potential and pH values caused, for example, by mixing with native groundwater (e.g., carbonate precipitates, iron hydroxides) (Bouwer, 2002; Caldwell, 2006). Sediment accumulation in the pores can lead to chemical clogging of the aquifer and the nearby intake well (Schubert, 2002).

The deposition rate of suspended particles, the size distribution of fine particles, and the size dispersion of receiving sediments are among the most critical three elements that determine the clogging rate of RBF systems, as well as the amount of clogging itself. According to Locke et al. (2001), the vertical distribution of fine particles in porous media is crucial because the main barrier to clogging in testing is the typical depth of penetration and deposition of particles. Recognizing how particles are distributed along the profile of porous media is essential for assessing the speed and depth at which clogging might occur. When it comes to solving the problem of blockages in riverbeds, it is necessary to consider several aspects, including the settling rate of suspended particles, the size distribution of fine fractions, and the characteristics of incoming sediments.

Moreover, comprehending the vertical distribution of fines within porous media is vital for predicting the degree and depth of clogging in RBF systems. This understanding is crucial in designing and managing RBF systems to ensure long-term sustainability. Hubbs (2006) highlighted that the main controlling factors of clogging include surface water quality, river hydrology, riverbed characteristics, and the positioning of the well concerning discharge and distance from the riverbank. Grischek and Bartak (2016) suggested that sites with a river flow velocity exceeding 0.8 m/s are recommended, and caution should be exercised to avoid excessive water abstraction from a restricted area.

### Water quality assessment during the infiltration process

Both bank filtration and artificial infiltration can be used as primary water treatment methods (especially in developing countries) and as pre-treatment methods for advanced methods, such as membrane treatment, activated carbon adsorption, and biofiltration (in high-income nations). They reduce or remove pollutants like organic matter, turbidity, surfactants, and pharmaceuticals present in natural surface water. Water quality characteristics are significantly changed by processes that take place in the soil during the infiltration process.

RBF is a natural technique that improves surface water quality, often in rivers, by using an aquifer connected to surface water and wells to extract water. This method involves using both a riverbed and an aquifer to lower the concentrations of dissolved and suspended contaminants in surface water. The process of riverbank filtration includes physical filtering, sorption, and degradation, which significantly reduces the number of pollutants, including physicochemical and microbiological contaminants. Samples were taken and analyzed from several parts of the RBF site, including surface water and pumping wells, to assess the water quality. The efficiency of the BRF process can be estimated by comparing the quality of the samples. A study done by Kim et al. (2013) indicated that BOD, COD, SS, and T-P contaminants were removed by 50%, 52%, 12.1%, and 52.4%, respectively. Generally, water collected from the wells after infiltration is substantially cleaner than river water (Akgiray & Soyer, 2006).

**Fig. 9:**
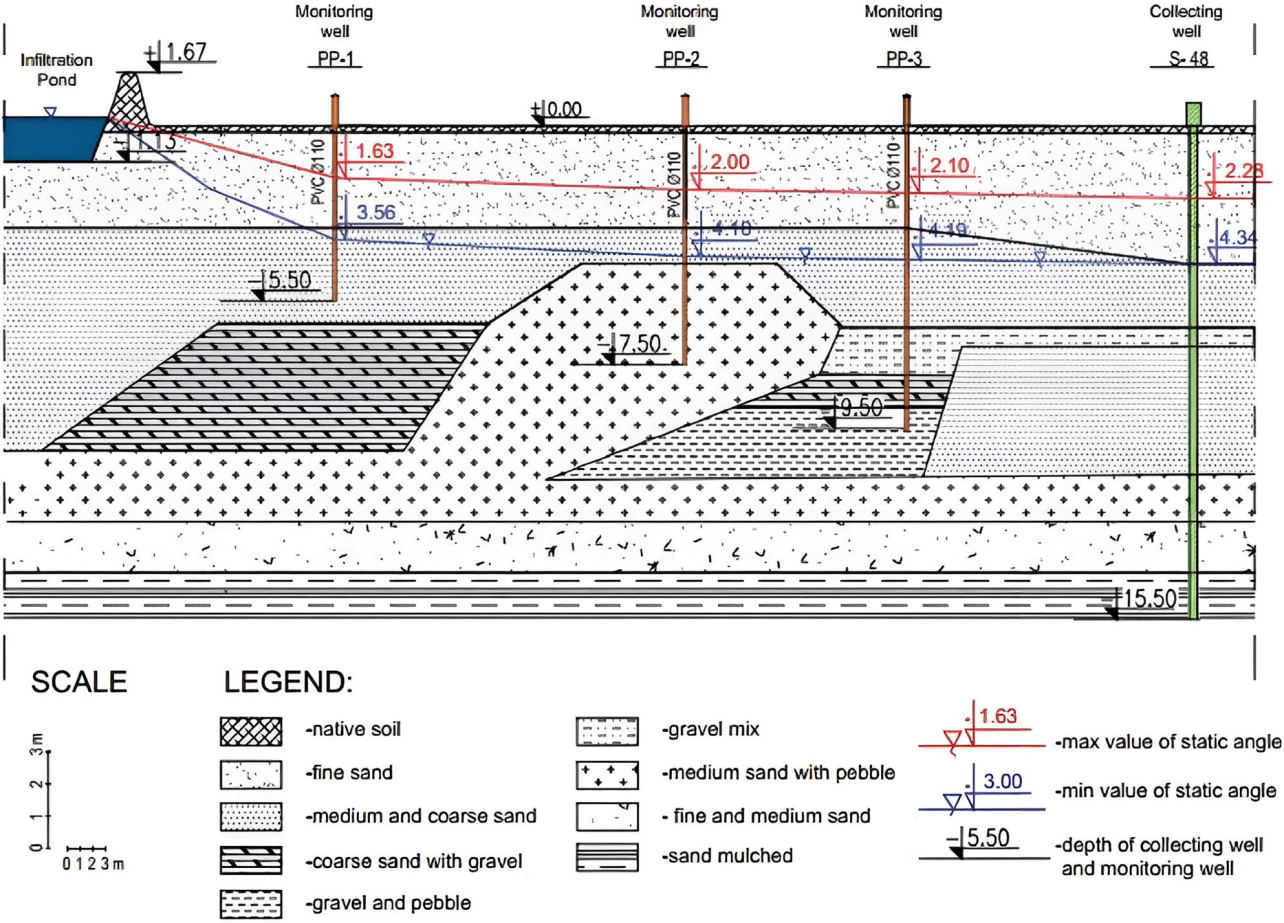
The cross-section of the infiltration path (Cierniak et al., 2020)

The study conducted at the artificial infiltration intake in Poland proved the high effectiveness of artificial infiltration. Untreated river water is first pumped out to the infiltration ponds. The experimental train (sampling points) consists of surface water (river and pond) and groundwater (piezometers and wells). Water passes from the bottom of the infiltration pond via piezometers before reaching the well (Fig. 9) (Cierniak et al., 2020). A significant decrease in biological and physicochemical parameters in well water compared to surface water was observed (Jeż-Walkowiak et al., 2021). In short, both infiltration types are robust and promising solutions for water treatment in low-income countries.

#### Turbidity removal

Turbid waters that are a large group of microbes and pathogens mark suspended solids (SS). Ray et al. (2002) showed that an association of underground processes such as adsorption, biodegradation, and straining with bank filtration successively removes or decreases particles. Furthermore, the efficiency of turbidity removal through the riverbank filtration (RBF) process has been approved by several researchers, such as Dash et al. (2008) and Saini et al. (2013). Numerous studies on turbidity removal using riverbank filtration (RBF) have been conducted on various continents involving different rivers, as illustrated in Table 3.

**Table 3:** Comparison of turbidity removal by RBF in the different rivers

Name of the country/city	Name of river	Turbidity of source water	Turbidity removal by RBF	References
USA/Washington	Columbia		1.9 log	(Mikels, 1992)
	Kalama	1–5 NTU	0.3–0.4 NTU (1.1 log)	(Mikels, 1992)
Egypt	Nile		2.5–0.25 NTU	(Abogabal et al., 2020)
India/Haridwar		1.9–2745	0–1 NTU (1 log non monsoons; 2-log monsoons)	(Dash et al., 2008)
USA/Jeffersonville	Ohio	60.1 NTU	0.1–0.2 (2.5–2.8 log)	(Weiss et al., 2005)
USA/Terre Haute	Wabash	190.3 NTU	0.1 (3.3 log)	(Weiss et al., 2005)
USA/Parkville	Missouri	78.6 NTU	0.5 (2.2 log)	(Weiss et al., 2005)
Indiana/Haridwar	Ganga	200–2500	0.6 NTU (99 ± 99.9%)	(Thakur & Ojha, 2010)
Colombia/Puerto Mallarino	Cauca River	Up to 700 NTU	35 NTU (95 ± 97%)	(Gutiérrez et al., 2017)
Brazil/state of Santa Catarina	Itajaí-Açu River	83 NTU	Less than 1 NTU	(Emmendoerfer et al., 2021)

Nevertheless, the efficiency of removing suspended solids varies based on their concentration, especially in cases where high turbidity indicates the presence of SS (Thakur & Ojha, 2010). A higher concentration of suspended particles, which in turn leads to increased rates of turbidity clearance, is responsible for the fast creation of the cake layer of accumulated suspended solids. That layer is formed on the river or pond bed during filtration processes. It reduces the permeability of the riverbed, thus hindering the infiltration and flow of water.

#### Nutrient removal

Different forms of nitrogen include nitrite, nitrate, and ammonium. Studies have shown that these forms can change due to denitrification or nitrification (Bai et al., 2023a, 2023b). According to the study conducted by Buzek et al. (2012), indeed, nitrate concentration decreased significantly due to nitrification, which is influenced by the distance and time it takes for river water to reach RBF extracting wells. Ammonium concentration is generally lower in surface water than treated water due to river nitrification processes. In the unsaturated conditions of the riverbank filtration (RBF) process, combined with oxic (oxygen-rich) conditions, reports suggest a decline in ammonium levels and a rise in nitrite and nitrate concentrations (Wu et al., 2007). According to reports, more than 95% of nitrogen removal occurs through nitrification and denitrification under saturated conditions. Meanwhile, phosphorus removal primarily occurs through sorption and precipitation processes (Regnery et al., 2015). As per Kaetzl et al. (2020), the primary influencing factor for phosphorus removal is the sedimentary structure of the subsoil.

#### Heavy metal removal

Ion exchange, sorption, and precipitation processes in underground passages are the main influencing factors of heavy metal retention. Heavy metals may precipitate as sulfide under anaerobic conditions (Schmidt et al., 2003). The study conducted by Bordas and Bourg (2001) confirmed that despite RBF’s potential for site- and substance-specific removal, it is a suitable water treatment method for removing heavy metals. Many RBF systems can remove heavy metals, such as arsenic and chromium, with an efficiency of nearly 90% (Schmidt et al., 2003; Stuyfzand et al., 2006). There have been observations of comparable results in removing heavy metals using various treatment methods, such as sand filtration (Kubier et al., 2019). Schmidt et al. (2003) reported that water from the Rhine River in Germany underwent RBF treatment, effectively removing around 75% of lead and cadmium.

In a study conducted in Germany, an RBF system was found to effectively remove various elements such as zinc, copper, lead, nickel, chromium, tin, arsenic, cadmium, selenium, silver, mercury, and cobalt with removal rates ranging from few to 94% over 3 years. Moreover, heavy metals introduced through surface water are readily eliminated, whereas those originating from geogenic activities pose a more significant challenge to control. Research documented a noteworthy decrease in Fe^3+^ and Mn4 + levels and increased Fe^2+^ and Mn^2+^ concentrations in the bank filtrate as it traversed the bank filtration process (De Vet et al., 2010; Gross-Wittke et al., 2010). According to Gross-Wittke et al. (2010), due to the increased microbiological and respiratory activity brought on by a decrease in redox potential and a rise in surface water temperature, iron and manganese were reductively dissolved at a bank filtration site in Berlin, Germany. However, Stuyfzand et al. (2006) showed a notable increase in lead and cadmium concentrations, rising by more than 300% and 30% in wells abstracting water within 450 days.

#### Micro-pollutant removal

Organic micropollutants (OMPS) released into water affect the health of all forms of life (soil and aquatic) on the land and in the environment. Most surface water that passes through large industrial and agricultural areas is likely to be polluted by micro-pollutants, worsening environmental and human life issues (Kondor et al., 2020). Hollender et al. (2018) indicated that RBF can impair OMP, and Sandhu et al. (2019) revealed that 13–99% of micro-pollutants were removed along the Yamuna River in India at RBF sites. The physicochemical properties of OMP are the main factors influencing their consequences. According to the European Framework Directives (EFDs) and the United States Environmental Protection Agency (USEPA), several OMPS have been designated as surface and groundwater contaminants. Despite this, RBF is effective in removing organic micropollutants. However, further treatment processes are recommended on the other side, where RBF is not the best choice and is mainly used to remove contaminants with higher hydrophobic and aromatic properties, including carbamazepine and ibuprofen (Bertelkamp et al., 2014). Based on the study conducted by Betancourt et al. (2019), many of the pharmaceuticals and personal care products evaluated were successfully removed except carbamazepine and primidone.

Sudhakaran et al. (2013) examined different water treatment approaches such as RBF, nanofiltration, reverse osmosis, adsorption, advanced oxidation, and ozonation to remove OMPS by considering different factors, including technical aspects, amenability, long-term economic viability, and time. Ultimately, they decided that RBF became the efficient and effective method of OMPS removal compared to membrane and adsorption processes. Residence time and redox conditions are the main factors of the biodegradation process of organic micropollutants. Schmidt et al. (2004) stated that the degree of elimination of any micropollutant relies on different factors, including local geological and hydro-chemical conditions and surface water and infiltration zone organic content.

According to the study of Abogabal et al. (2020), RBF has the dexterity to remove micro-pollutants, including pharmaceuticals, and Kondor et al. (2020) agreed that RBF can efficiently remove pharmaceutical compounds. Gross-Wittke et al. (2010) proposed that the removal of pharmaceuticals can be achieved through adsorption, and biodegradation takes place during the percolation process. Temperature influences the elimination of micro-pollutants, with higher temperatures potentially enhancing the adsorption properties of organic micropollutants. Additionally, increased temperatures stimulate biological activity within the sand, accelerating the biodegradation rate of compounds and resulting in higher efficiency in micro-pollutant removal. Under anaerobic conditions, some micropollutant, including sulfamethoxazole, was primarily removed for approximately 20%, while under aerobic conditions, the efficiency removal was less than 20% (Schmidt et al., 2003). The study conducted by Makała et al. (2021) has confirmed that pharmaceutical drugs such as paracetamol go down progressively after water passes through infiltration. In addition, the biodegradation process that occurs when water moves through the soil is essential in eliminating surfactants (Makała et al., 2023).

## Factors influence the workability of bank filtration

### Raw water quality

Evaluation of raw water quality is crucial in the design and operation of RBF wells. The water generated by the RBF process is affected by several different elements, such as the hydrogeology of the site, the type of well and its location, the biogeochemical processes taking place, the rate of depletion of streams, and the quality of the source water (Gillefalk et al., 2018; Rao et al., 2020).

### Traveling time and redox conditions

The amount of surface water available for the RBF process can be affected by changes in severe weather conditions. For this reason, river flow times are affected. Longer travel times are observed during low water levels, while shorter travel times occur during floods. The time the water is forced to travel underground and the redox conditions it encounters throughout its journey are two of the most critical aspects that influence how efficient RBF is at removing impurities. The properties of the aquifer, in addition to the quality of the raw water, have a significant effect in determining the redox conditions that affect the environment. The trip time of riverbank filtrate is a crucial aspect that plays a role in determining the efficacy of RBF systems in removing substances (Kuehn & Mueller, 2000). The increase in travel time positively affects contaminant removal and increases the prospect of environmental anaerobic conditions (Schijven & Hassanizadeh, 2000). In addition, van Driezum et al. (2019) have agreed that a bank filtrate with low-velocity travel time is more favorable than a short time. The quality of bank filtrates is improved due to that correlation, which can help reduce unnecessary and toxic elements such as iron, manganese, and arsenic. According to different literature sources, a travel/residence time of 10 to 50 days was estimated to be admissible (Abdelrady et al., 2020).

Water quality parameters vary with changes in redox condition. For instance, a study conducted at Elbe River (Germany) and Warta River (Poland) revealed an increase in sulfate in infiltrating water. This phenomenon occurs due to changing the reduction condition to an oxidation condition caused by aerated water flow and abstraction. After the change of redox condition, sulfides present in the ground (the product of organic matter degradation) are oxidized to the form of sulfates (Górski, 2011). Conversely, iron and manganese concentrations increased due to the blending of the RB filtrate with groundwater. With increased travel distance, there was a lower ammonium concentration at the Warta River. Górski (2011) proposed that a travel distance of either 150 or 250 m could result in improved water quality during the RBF process at the Warta River. In another research (Ray et al., 2002), it was revealed that since the pumping well is much closer to the river, the higher the hydraulic conductivity, the higher the permeability of the water, which will help contaminants to enter quickly, thus causing a large amount of low-quality water on the other side, causing the inverse.

The duration of travel or residence time significantly influences contaminant removal efficiency, surpassing the impact of route length. This occurs when residence times are distinct while the travel distance remains the same, considering the hydrogeological conditions at the site and the properties or behaviors of surface water included in the RBF scheme. Consequently, travel or residence time can be used to predict water quality. Conversely, when the residence time results are unavailable, travel distance is an alternative approach to predicting removal efficiency. The time it takes for water to move from the banks to the production well is a significant factor contributing to the effectiveness of contamination removal in many situations. Several elements often affect the expenses involved in the RBF system. These considerations include the characteristics of the aquifer, the type of well screen installation, the architecture of the facility, and the distance from the population served. In addition, the cost of operation and maintenance is evaluated or estimated according to several factors, including the size of the bank filtration system, the types of wells that are implemented, whether they operate continuously or intermittently, the materials that are used for the building of the wells, the geological environment, and the state of the river (Hunt & Stabeno, 2002). Adjusting the separation from the bank and the spacing between wells allows one to get the required water amount and quality. This modification meets certain parameters to maximize well performance, such as permitted downgrades and production capacity (Sharma et al., 2012).

### Operation during droughts and floods

An important issue is to estimate the operating conditions of the intake in the event of a hydrological drought in the intake area. The drought period affects the efficiency of the RBF intake and that supplied via infiltration ponds. During a drought, the efficiency of the intake can be reduced and even the hydrological continuity can be interrupted. Hydrological models make it possible to determine the capacity of an infiltration intake during a drought that causes an interruption in the infiltration ponds and to what extent the capacity will change when the surface water supply is interrupted and only groundwater and infiltration resources in the ground can be used during this time. Infiltration intakes are characterized by some retention of infiltrating water in the aquifer, which, depending on hydrological conditions, can provide a supply of water from several days up to several weeks.

During floods, the quality of surface waters may deteriorate, which affects the quality of infiltration waters. The deterioration of infiltration water quality may occur due to the deterioration of river water quality, substantial shortening of groundwater passage, and partial disappearance of the aeration zone. Particularly important is the risk of microbiological contamination of infiltration waters during floods due to possible contamination of flooded wells and shortened infiltration route.

## Impact of climate change on surface water quality and infiltration performance

Climate change substantially impacts the RBF system’s functioning, particularly in terms of temperature and precipitation (Skaland et al., 2022), changes in streamflow, droughts, and water scarcity. The significant acceleration of climate change directly affects the availability of surface water, which directly contributes to disasters such as floods and droughts. Alterations in temperature and precipitation significantly influence the amount of surface water available, affecting the RBF system’s capacity to function qualitatively.

There is a reciprocal relationship between temperature and precipitation, which means that both factors affect the overall functioning of the RBF system. Rising temperatures can lead to increased water temperature, affecting the dissolved oxygen levels and promoting the growth of harmful algae and bacteria. Changes in precipitation patterns can result in more frequent and intense storms, leading to the runoff of pollutants into water sources. This runoff can cause contamination, increasing the risk of waterborne diseases (Herrador et al., 2016). Changes in precipitation patterns and the melting of glaciers can lead to variations in streamflow, impacting both water availability and the dilution of pollutants. Climate change can worsen drought conditions, leading to low river water levels, further reducing water availability and resulting in higher concentrations of contaminants in surface water (Eckert et al., 2008; Samperio et al., 2021).

Climate fluctuation in Africa is famous. The main contributing factor to floods and droughts is the variation of precipitation. However, in sub-Saharan Africa, droughts are the main climatic danger. They endanger lives and food and water security, significantly impacting GDP growth in one-third of the continent’s nations (Stavi et al., 2021). The RBF system is greatly affected by climate change (Nagy-Kovács et al., 2018). Due to their susceptibility to severe weather events, RBF processes have recently become the focus of increased research on the effects of climate change (Sprenger et al., 2011). The quantity and quality of filtration on riverbanks can be affected by floods and droughts resulting from climate change, subsequently affecting the efficiency of the filtration process (Delpla et al., 2009). Assessing and effectively managing the impact of extreme weather conditions is critical to meeting the challenges of ensuring clean water availability.

During drought, the amount of suspended solids and sediment increases, which can lead to clogging of the riverbed. On the other hand, flooding is a shear force that can help the riverbed clean itself by removing deposited wastes (Hiscock & Grischek, 2002). There is a reciprocal relationship between temperature and precipitation, meaning that both factors affect the overall performance of the RBF system. Drought conditions associated with high temperatures (lower oxygen solubility) can intensify the occurrence of anaerobic conditions during the filtration process by reducing the flow of oxygenated water. This, along with the subsequent effects of flooding reducing travel time, reduces the purification effect of infiltrating water (Sprenger et al., 2011).

The Nordic regions are projected to face future challenges from climate change, such as significant precipitation and rising temperatures. These changes will significantly affect the levels of pathogenic bacteria and NOM in surface water, which will change the interaction between surface water and the aquifer, reduce the availability of river water, and ultimately affect the implementation of the BF. In addition, Beyene et al. (2010) found that surface water availability in the Nile River basin is predicted to decrease due to climate change and falling precipitation levels. This decrease could affect travel time, reducing BF performance.

Climatic conditions also affect the quality of bank filtrate. During floods, reduced residence time of water in the ground is a major factor in reducing the effects of the infiltration process. Flooding can also lead to higher sediment loads in the water, which can clog the filtration pathways in the aquifer, reducing the overall efficiency of the bank filtration process (Grischek & Bartak, 2016; Pholkern et al., 2015). Additionally, floodwaters may contain various contaminants, including nutrients and pathogens, that are washed into the river system, further degrading the quality of the bank filtrate (Gillefalk et al., 2018). The quality of filtrate is also affected by the increased frequency and intensity of floods. High rainfall, for example, can cause increased runoff that carries pollutants from the surface into the aquifer, introducing different contaminants that affect the filtrate quality. Additionally, warmer temperatures can create ideal conditions for microorganism growth, leading to higher biological activity in the aquifer and increased levels of bacteria, viruses, and other pathogens in the filtrate. Ultimately, the rise in sea level resulting from climate change has the potential to lead to saltwater intrusion into freshwater aquifers. This intrusion can further threaten the quality of bank filtration.

Ascott et al. (2016) indicated that dissolved oxygen (DO) increased by an average of 4.1 mg/L following flood. In June 2014, the average DO concentration was 0.93 mg/L. Therefore, after flooding, the DO concentration increased more than fourfold. This is due to direct infiltration of floodwater, rapid recharge from rainfall, and a reduction in the unsaturated zone as the groundwater level rises. In addition, the landfills located near the RBF system may contribute to the increase of dissolved organic carbon (DOC) in the aquifer. On the other hand, infiltration ponds located at the intake may have higher levels of turbidity, organic matter, and microbial contamination during floods.

The effectiveness of infiltration in removing some contaminants, including turbidity and microbiological properties, requires about 6 weeks to return to pre-flood conditions. Further withdrawal of infiltrated water introduces contaminated flood water into the groundwater system. The permeable materials of the aquifer allow contaminants in a saturated state to be carried into the well and quickly recharged. These issues later affect the quality of the riverbank filtrate. Drought conditions can lead to a significant reduction in the volume of water stored in aquifers, which concentrates existing pollutants and reduces the natural dilution effect that typically helps maintain water quality (Nie et al., 2018). As water levels decrease, contaminants such as nitrates and heavy metals may become more concentrated, posing greater risks to human health and ecosystems (Gillefalk et al., 2018). Additionally, lower water levels can prolong the residence time of water within the aquifer, allowing for more prolonged interactions between water and contaminants (Nie et al., 2018). This increased contact time can facilitate the leaching of pollutants from surrounding materials into the water (Gillefalk et al., 2018). Consequently, drought conditions worsen the overall degradation of water quality, underscoring the critical need for effective water management strategies during such periods.

Climate has the potential to have a significant impact on the amount of bank filtrate that is produced. Climatic factors such as temperature, precipitation, and evapotranspiration rates directly influence the hydrological cycle, affecting both groundwater recharge and river flow patterns (Kundzewicz et al., 2008). It has been observed that there was an increase in the amount of water that seeped into the collector well, and water levels were higher. However, extreme weather conditions like intense rainfall or drought can intensify fluctuations in river levels, altering the filtration rates significantly (Hiscock & Grischek, 2002).

## Monitoring of operational parameter of infiltration intake

The techniques for monitoring the main operational parameters are important for the control of the effectiveness of water intake. A detailed explanation of each technique is provided below.

### Residence time monitoring

To monitor residence time, the tracer technique is applied, which involves introducing a conservative tracer (e.g., salt or dye) into the water source and tracking its concentration downstream. This method allows for the calculation of residence time based on the movement and dilution of the tracer. Additionally, employing flow metering techniques such as electromagnetic or ultrasonic flow meters can provide continuous measurements of flow rates, which can be critical in estimating residence time based on volume and flow rate. Furthermore, using hydrological modeling, such as MODFLOW, can simulate groundwater flow dynamics and assist in understanding residence times in the system (Ehtiat et al., 2018; Putra et al., 2022). The simple and effective way to evaluate the residence time is frequent monitoring of the change of infiltrating water temperature in metering wells (piezometers located on the way of water ground passage). The wave of the highest and the lowest temperature moves across the water path, indicating the time necessary to travel between the measuring point (Makała et al., 2023).

### Quantifying bank filtrated water

For quantifying the amount of bank-filtrated water, a combination of hydraulic gradient measurements and water quantity measurements is recommended. Installing piezometers to measure groundwater levels and gradients helps to assess the groundwater flow. The test pumping allows to estimate the well capacity. Using remote sensing techniques, including satellite imagery and aerial surveys, can also provide valuable insights into surface water dynamics and help estimate the volume of bank-filtrated water (Labelle et al., 2023; Ramli et al., 2017).

### Monitoring clogging development

Monitoring clogging development is essential for maintaining system efficiency. One effective approach is to use pressure drop (head loss) measurements across the infiltration path. Installing pressure sensors can detect changes in pressure indicative of clogging, providing a quantitative understanding of the filtration performance. Regular visual inspections and sampling (of sediment on the bottom of a river or pond) can complement this approach, allowing for the examination of microbial and particulate buildup within the filtration media. Additionally, implementing automated monitoring systems that continuously record parameters like flow rates and pressure drop can offer real-time data on clogging, enabling timely maintenance actions (Pholkern et al., 2015; Ulrich et al., 2015). The drop in collecting well capacity also indicates the clogging occurring in the ground around the well. This clogging is of chemical origin.

### Water quality monitoring

Regular water sampling from piezometers installed all around the area of water intake and analysis of water quality parameters allow to estimate the effectiveness of water purification at different parts of the intake area. The use of automated water quality sensors for continuous monitoring can enhance our understanding of changes in water quality over time, providing critical data for operational management (Rahim et al., 2017; Zainurin et al., 2022).

### Flow dynamics

Understanding flow dynamics is integral to managing water resources effectively. Employing hydraulic models alongside flow monitoring equipment can help analyze flow patterns and dynamics within the system. Such assessments are crucial for optimizing flow management strategies and identifying potential areas for improvement (Mashaly & Fernald, 2020).

## Current limitations and efficiency of riverbank filtration

Constraints related to dynamics (transport, runoff, erosion, and sedimentation) and surface and groundwater hydrology should be taken into account when evaluating the efficiency and effectiveness of the RBF system (Ray & Prommer 2006). The limitations of RBF can be summarized as follows: changes in the hydraulic gradient between river water and the aquifer may lead to retention time and pore water velocity alterations, influencing biogeochemical processes in the hyporheic zone. Factors such as variations in river stage, biofilms content, water saturation, geochemistry, and the structure of the RBF system can impose constraints on the treatment’s efficacy. These variations can impact flow and transport dynamics, as well as the overall efficiency of the RBF process. The divergence in contaminant removal potential between saturated and unsaturated zones contributes to these limitations (Singh et al., 2010). High flow rates can impact the effectiveness of the riverbank filtration (RBF) process by influencing scouring processes during filtration (Gollnitz et al., 2004).

Scouring processes primarily result in the loss of fine sediments, which can reduce the permeability of riverbeds. Additionally, it leads to removing microorganisms crucial for improving the hyporheic zone’s water quality (Bai, Jing, et al. 2023). A common issue in RBF is the potential clogging of the aquifer due to aggregation of suspended solids screened during river/lake water infiltration, especially in cases of insufficient system design. Key factors influencing clogging include river dynamics (transport, runoff, erosion, and sedimentation), freshwater conditions, healthy river morphology placement, and the distance between the well and the riverbank. Riverside filtration (RBF) is dependent on site conditions, making it a viable and practical option when favored by local factors such as geography, hydrogeology, hydrology, soil texture, water quality, and cost (Hiscock & Grischek, 2002; Lee & Lee, 2010). However, while riverbank filtration (RBF) depends on local conditions, the availability of surface water is a fundamental and key element of the RBF system. Accordingly, the system is designed to improve surface water quality, complementing existing recharge systems (Tyagi et al., 2013). Caldwell (2006) have successfully implemented riverbank filtration in rivers with average discharges ranging from 20 to 3000 m^3^/day and surface water slopes ranging from 0.04 to 1.8 m/km.

## Riverbank filtration vs conventional treatment and cost

**Fig. 10:**
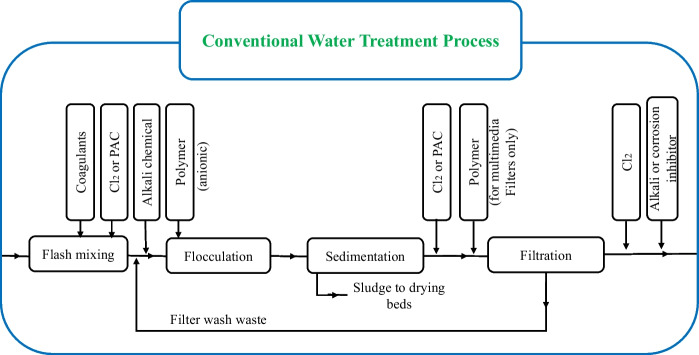
Conventional water treatment process

**Fig. 11:**
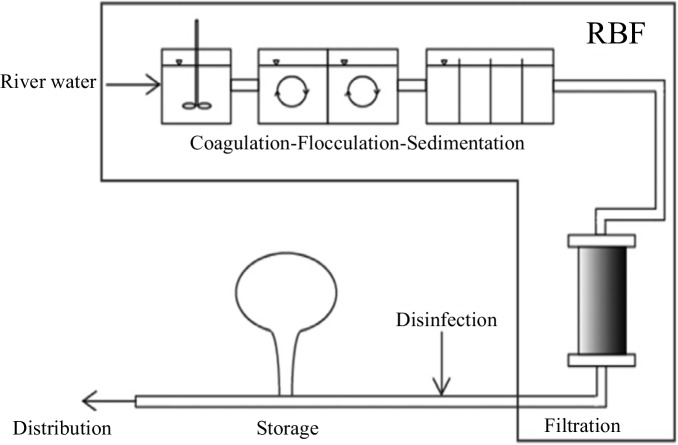
Combination of conventional water treatment process vs RBF

RBF is a low-cost, reagents-free, so eco-friendly water treatment technique. In contrast, conventional water treatment methods (Fig. 10) are based on physical–chemical processes requiring dosing of several chemical reagents. The use of chemicals not only increases costs but also impacts the system’s operation and maintenance. Systems that use fewer chemicals tend to generate less sludge. Sludge management involves equipment and disposal issues. Inhibiting these and other factors relates to sustainability. RBF in comparison to conventional water treatment has various advantages in terms of energy costs (Fig. 11) (Jaramillo et al., 2011). As RBF counts on natural filtration processes, it requires less energy and equipment, thus lowering costs compared to conventional treatment methods. It is also a sustainable method due to its reliance on natural processes, avoiding the use of chemicals and reducing the environmental impact of the water treatment process.

The study conducted in developing countries, especially in Africa (Malawi and Kenya), displayed that more than 80% of annual treatment costs (for only chemicals and energy) can be saved by shifting the RBF system from conventional water treatment in Blantyre and Lilongwe cities, Malawi. Although chlorination is still used after the RBF process, there is potential for savings through the use of the riverbank than conventional water treatment in various cities in Malawi, such as Blantyre, Lilongwe, and Mzuzu, by 20%, 53%, and 2%, respectively. In various Kenyan cities, specifically Eldoret and Nakuru, transitioning from traditional surface water treatment to the BF system could result in annual operational cost savings of approximately 16% and 32%, respectively. Moreover, in Eldoret and Nakuru, the overall cost of water per cubic meter (m^3^) from the BF system is roughly half that of water produced through conventional water treatment systems.

Regarding capital expenses, the bank filtration system is anticipated to be sevenfold more cost-effective for Eldoret and four times more economical for Nakuru than conventional water treatment systems. BF and traditional treatments, such as slow sand filtration, are worth the comparison. Besides, the removal/decrease of DOC, particulate matter, microbial parameters, and pathogens by BF and biopolymers, proteins, and polysaccharides by slow sand filtration indicates minimizing membrane fouling. Otherwise, bank filtration reduces chemical use; thus, it is more profitable than sand filters. BF can remove the most insistent toxins as well as pathogens. Therefore, it can replace the existing conventional treatment techniques or be used as a suitable pretreatment for advanced treatment methods, such as membrane filtration for some high-resisting contaminants that are not removed by themselves (Baghoth et al., 2011; Maeng et al., 2010).

Factors such as the type of intake constructed and the necessary excavation work can also impact the cost of RBF. Despite these variables, RBF remains a promising option for areas with abundant natural water resources and pre-existing infrastructure. It is important to consider all costs, including construction, operations, and maintenance when evaluating the feasibility of RBF as a treatment solution. As demonstrated in a study conducted in Aswan City, Egypt, RBF technology can be more economically feasible than other treatment options. Table 4 shows the net present value (NPV), payback period (PBP), water treatment plant (WTP), and bank filtration (BF) (Abdelrady et al., 2020). The values in the table indicate the comparison of the cost for bank filtration with the conventional water treatment methods. However, we can conclude that RBF should be considered as a first option in low-income countries with suitable hydrogeological locations.

**Table 4:** Economic comparison between current treatment techniques and BF in Aswan City, Egypt

	UNIT	BF	GW	Low-capacity WTP	High-capacity WTP
Production capacity	m^3^/day	2160	2160	2160	8640
Capital cost	Million EGP/unit	0.5	0.8	5.0	35.0
Chemicals, operation, and energy cost/year	Million EGP/unit	0.5	0.5	1.0	1.8
NPV	Million EGP	6.2	5.9	0.9	7.6
PBP	Year	0.5	0.7	8.2	6.3

## The current state and outlook of RBF

Numerous studies, including those of Ray et al. (2002) and Tufenkji et al. (2002), have demonstrated that riverbank filtration has been used for over a century and continues to be applicable in various European regions. Presently, it is extensively used in both North and South America, as well as in Asia. Despite the feasibility studies conducted in two African countries, namely Malawi and Kenya, riverbank filtration is not widely implemented in developing countries (Sharma & Amy, 2009). Table 5 illustrates the global applicability of RBF in various regions, where it substantially contributes to potable water supplies in different countries (Gillefalk et al., 2018). Local circumstances are among the most critical factors determining RBF’s effectiveness. These variables include the hydrology and hydrogeology of the location, the geochemistry of the water (both from the river and the aquifer), the geochemistry of the microbial communities, and the metabolic activity linked with them.

**Table 5:** Percentage of bank filtration in the drinking water supply of different countries/cities and source surface water bodies

Country	City	Percentage of drinking water provided by IBF	Source water bodies	River discharge
France	Paris region	*	Seine River	450 m^3^/s
Germany	Berlin	60	-	-
”	Radeburg	-	Radeburg Reservoir	0.35 km^2^; max depth: 3 m
”	Düsseldorf	~ 100	River Rhine	2300 m^3^/s
”	Frankfurt am Main	*	River Rhine	2300 m^3^/s
”	”	*	Lower River Main	193 m^3^/s
”	Torgau and Mockritz	*	Elbe River	700 m^3^/s
Hungary	-	45	-	-
”	Budapest	-	Danube	6460 m^3^/s
”	*	-	Rivers Raba, Drava, Ipoly, Sajo, Hernád	17, 500, 21, 67, 27 m^3^/s
Italy	Lucca, Pisa, Livorno	(300,000 inhabitants)	River Serchio	46 m^3^/s
Norway "	Hemne "	* *	River Buga	*
Poland	Poznań	*	River Warta	60 m^3^/s
Romania	Iasi	*	Moldova River	143 m^3^/s
Slovak Republic	-	50	-	-
Slovenia	Maribor	-	Drava River	500 m^3^/s
Switzerland	-	10–30	- River Thur	- 40–50 m^3^/s
UK	*	*	Streams Wissey, Rhee and Pang	1.9, 1.25, 0.64 m^3^/s
USA	Jeffersonville	*	Ohio River	3512 m^3^/s
”	Santa Rosa	*	Russian River	66 m^3^/s
”	Cincinnati	*	Great Miami River	109 m^3^/s
”	Columbus	*	Scioto/Big Walnut Creek	6 m^3^/s
”	Galesburg	*	Mississippi River	16,792 m^3^/s
”	Independence Kansas City Parkville	*	Missouri River	2158 m^3^/s
”	Jacksonville	*	Illinois River	659 m^3^/s
China	Matan	96	Yellow River	1839 m^3^/s
India	-	*	-	-
”	Delhi	*	Yamuna River	100–1300 m^3^/s
Malaysia	Kuala Kangsar	*	Sungai Perak (river)	57 m^3^/s
South Korea	*	*	Nakdong River	37–3462 m^3^/s
Thailand	Chiang Mai	*	Ping River	287 m^3^/s
Egypt	-	0.1 (increasing)	Upper Nile	1548 m^3^/s
”	Sidfa	*	Nile	2830 m^3^/s
Brazil	Joinville, Santa Catarina	72	Cachoeira River	150 m^3^/s
Argentina	Buenos Aires	35	Río de la Plata and local rivers	-
Colombia	Bogotá	40	Bogotá River	30 m^3^/s

The symbol (") indicates that the information is the same as above, the asterisk (*) signifies that information is missing, and the dash (–) indicates that the information is not applicable

## Future perspective and conclusion

It is important to note that RBF is a treatment method highly dependent on local conditions. Based on current research, we can see various potential outcomes for RBF in the future. RBF is becoming increasingly important for sustainable water resources, as global population growth and water scarcity are significant concerns in many world regions. The method offers a promising solution by using natural processes to clean water without relying on adding chemicals or energy-intensive treatments. Additionally, RBF helps mitigate the impact of climate change by providing a reliable and resilient water supply compared to conventional methods. The method removes specific contaminants, such as suspended solids, pathogens, and organic compounds. In fact, future development may focus on enhancing the removal of pharmaceuticals, microplastics, and heavy industrial chemicals. Moreover, integrating RBF with other advanced treatment technologies, like membrane filtration or activated carbon adsorption, could improve overall water quality in the long term. RBF is an effective and efficient natural treatment method that inexpensively removes contaminants and expands quickly into new regions with suitable hydrogeological conditions.

## Recommendations

According to Ray & Prommer (2006) and Caldwell (2006), riverbank filtration should be located at the inner bends of the river, as it reduces the effect of aquifer clogging than the outer bends. Ideal sites for riverbank filtration (RBF) are characterized by sand and gravel aquifers with hydraulic conductivities exceeding 10^−4^ m/s, a minimum thickness of 5 m or a maximum of 10 m, and a robust hydraulic connection to the nearby surface water (De Vet et al., 2010; Tyagi et al., 2013). In addition, it may be located in an area consisting of an alluvial aquifer composed of silt and clay, which have low water permeability (Hubbs, 2006; Hunt & Stabeno, 2002; Sharma et al., 2012). Ascott et al. (2016) recommended that water managers support flexible operating systems, such as the one used in their study, to increase the potential of RBF systems during climate change-induced flooding. Recommended measures include regulatory flexibility to adjust pumping rates, implementation of variable speed drive pumps, infrastructure designed to withstand flooding, the establishment of adequate treatment, network, and storage capacity to manage increased water volumes, and adoption of appropriate treatment processes capable of dealing with different water properties. These measures collectively contribute to a comprehensive approach to enhance the efficiency and resilience of water management systems.

## Data Availability

No datasets were generated or analyzed during the current study.
